# Isolation of Cancer Stem Cells from Three Human Glioblastoma Cell Lines: Characterization of Two Selected Clones

**DOI:** 10.1371/journal.pone.0105166

**Published:** 2014-08-14

**Authors:** Fortunata Iacopino, Cristiana Angelucci, Roberto Piacentini, Filippo Biamonte, Annunziato Mangiola, Giulio Maira, Claudio Grassi, Gigliola Sica

**Affiliations:** 1 Institute of Histology and Embryology, Medical School, Catholic University of the Sacred Heart, Rome, Italy; 2 Institute of Human Physiology, Medical School, Catholic University of the Sacred Heart, Rome, Italy; 3 Institute of Neurosurgery, Medical School, Catholic University of the Sacred Heart, Rome, Italy; University of Torino, Italy

## Abstract

Cancer stem cells (CSC) were isolated via a non-adherent neurosphere assay from three glioma cell lines: LI, U87, and U373. Using a clonal assay, two clones (D2 and F11) were selected from spheres derived from LI cells and were characterized for the: expression of stem cell markers (CD133, Nestin, Musashi-1 and Sox2); proliferation; differentiation capability (determined by the expression of GalC, βIII-Tubulin and GFAP); Ca^2+^ signaling and tumorigenicity in nude mice. Both D2 and F11 clones expressed higher levels of all stem cell markers with respect to the parental cell line. Clones grew more slowly than LI cells with a two-fold increase in duplication time. Markers of differentiation (βIII-Tubulin and GFAP) were expressed at high levels in both LI cells and in neurospheres. The expression of Nestin, Sox2, and βIII-Tubulin was down-regulated in D2 and F11 when cultured in serum-containing medium, whereas Musashi-1 was increased. In this condition, duplication time of D2 and F11 increased without reaching that of LI cells. D2, F11 and parental cells did not express voltage-dependent Ca^2+^-channels but they exhibited increased intracellular Ca^2+^ levels in response to ATP. These Ca^2+^ signals were larger in LI cells and in spheres cultured in serum-containing medium, while they were smaller in serum-free medium. The ATP treatment did not affect cell proliferation. Both D2 and F11 induced the appearance of tumors when ortotopically injected in athymic nude mice at a density 50-fold lower than that of LI cells. All these data indicate that both clones have characteristics of CSC and share the same stemness properties. The findings regarding the expression of differentiation markers and Ca^2+^-channels show that both clones are unable to reach the terminal differentiation. Both D2 and F11 might represent a good model to improve the knowledge on CSC in glioblastoma and to identify new therapeutic approaches.

## Introduction

There is increasing evidence that tumors are hierarchically organized by heterogeneous populations including a small fraction of cancer stem cells (CSC). CSC share many similarities with normal stem cells, such as self-renewing capacity and multilineage differentiation properties [1]. In addition, CSC are highly tumorigenic and can generate phenocopies of the primary human malignancy in immunocompromised mice [1]. From a clinical point of view, CSC are responsible for tumor maintenance, sustentation, recurrence and resistance to conventional treatments [2]–[4].

A CSC fraction has been isolated in many cancers, including glioma [2]–[5], using various approaches [5]–[9]. Most glioma CSC have been derived from clinical tumor specimens [7], [10], [17] while only a few have been derived from established cell lines: Rat C6 cells and human malignant glioma cell lines (U373, A172, U87 and SU3) have been used [9], [17]–[23], [24]. Some Authors do not recommend cell lines as a source of CSC because they grow in serum containing medium, which gives rise to cells that differ genetically and biologically from those of the primary tumors from which they were derived [25]. Nevertheless, cancer cell lines have some advantages with respect to tumor tissue. Indeed, they do not present any contaminating normal stem cells, can be considered a homogeneous sample and it is easy to obtain large amounts of them [21]. Therefore, identification and characterization of CSC from established cell lines may provide important tools for exploring the biology of CSC [26]. No single marker has been shown to be sufficient to confer stem-cell-like properties, thus a combination of different markers is used to identify and isolate CSC in glioma, including Nestin, Sox2 (SRY-related HMG-box gene 2) and Musashi-1 (Msi-1). These molecules are expressed at high levels in neural stem cells and are frequently considered a hallmark of the undifferentiated state [27]–[30].

When exposed to fetal bovine serum, CSC differentiate down the lineage of the parental tumor [6], [9], [12], [16]–[23]. Therefore, CSC derived from gliomas preferentially differentiate to astrocytes, but multilineage differentiation can occasionally be observed with neuronal lineages, and some abnormal cells with mixed phenotypes. It should be noted that these lineages are characterized on the basis of molecular markers, such as the astrocytic marker GFAP, the oligodendrocytic marker GaLC, and the neuronal marker (βIII-Tubulin) [7], [9], [16]–[23], [25], rather than on functional parameters. For example, the crucial test to identify a neuron should be to assess its ability to generate action potentials [31], [32], but this test is not usually performed. Moreover, the important role of the Ca^2+^ signals in the development of glioblastoma (GBM) has recently been reviewed [33].

Some interesting results have been obtained using CSC derived from established cell lines regarding invasive properties, chemoresistance, drug screening, apoptosis, proliferation, immune responses, and gene expression [34]–[39].

In this study, we found that U87, U373 and LI cell lines contain a fraction of cells that can form tumor spheres when cultured in serum-free neural stem cell medium. Cells from tumor spheres possess the capability of self-renewal and secondary spheres formation.

It is well known that in GBM there is a histological variability and heterogeneity [40], [41] that results in the isolation of distinct CSC subpopulations which maintain the primary tumor phenotype and genotype, as recently described [19], [25]. Our results clearly show that all the human glioma cell lines we studied contain CSC. Moreover, two clones selected from the LI cell line were characterized for: expression of stemness markers, proliferation, ability to differentiate, presence of voltage-gated Ca^2+^ channels and ATP-dependent Ca^2+^ signals, and tumorigenicity after orthotopic transplantation. To our knowledge, no data are available in the literature about the presence of CSC in this LI cell line. These two clones may represent models useful for future studies on malignant gliomas with the aim to find new therapeutic approaches.

## Materials and Methods

### Cell lines

#### Culture of glioma cell lines

The human cell line U373 (American Type Culture Collection, ATCC, Rockville, MD, USA), astrocytoma grade III/glioblastoma, was kindly provided by Professor Pierluigi Navarra, Institute of Pharmacology, Catholic University of the Sacred Heart, Rome.

The human cell line U87 (ATCC, USA), astrocytoma grade III/glioblastoma, was kindly provided by Dr. Emilio Ciusani, National Neurological Institute “Carlo Besta”, Milan [42].

The human LI cell line, astrocytoma grade IV, was kindly donated by Professor Gabriella Zupi, Regina Elena Institute, Rome [43], [44].

The three cell lines (parental cell lines) were cultured in Dulbecco’s modified Eagle’s medium/Ham’s F-12 (DMEM/F12) (Gibco, Carlsbad, CA) containing 10% fetal bovine serum (FBS, Euroclone, Milan, Italy), antibiotics (penicillin/streptomycin 100 IU/ml/100 µm/ml, Euroclone), 4-2-hydroxyethyl-1-piperazinyl-ethanesulfonic acid (10 mM, Euroclone), glutamine (2 mM, Euroclone) and glucose (0.3% w/v, Sigma-Aldrich, St Louis, MO, USA), hereafter named: standard medium. The cells, which grow in monolayer, were subcultured to confluence, and maintained at 37°C in humidified atmosphere air: CO_2_ (95%: 5%).

### Neurosphere (NS) culture

All the monolayer growing cell lines, U373 (22–25^th^ passage), U87-MG (52–55^th^ passage) and LI (93–95^th^ passage) were seeded at a density of 100,000 cells/ml, in a defined serum-free medium, DMEM/F12, supplemented with antibiotics (penicillin 100 IU/ml, streptomycin 100 µm/ml), HEPES (10 mM), glutamine (2 mM), glucose (0.3% w/v), recombinant human epidermal growth factor (rhEGF) (20 ng/ml, R & D Systems, Minneapolis, MN, USA), basic fibroblast growth factor (bFGF, 20 ng/ml, R & D Systems) and N-2 Plus Media Supplement (R & D Systems) (Neural Stem Cell Medium, hereafter named: NSCM). The medium was changed every 2 days. Spheres were split by mechanical dissociation when they reached a size of 100–200 cells.

### Limiting Dilution/Tumor Sphere Initiation Analysis

A Limiting Dilution/Tumor Sphere Initiation Analysis was performed to quantify, in the three parental lines, the frequency of Tumor Spheres Initiation cells (TS-ICs) according to the method described by Yu A et al. [22]. The percentage of TS-ICs and statistical significance were determined by using the L-CALC (Stemsoft, Vancouver, Canada), a freeware software program http://www.stemcell.com/Default.aspx.

### Single colony formation analysis

Mechanically dissociated cells were seeded into 96-well plates at a theoretical density of 0.5 cell per well in NSCM. After overnight culture, microscopic observation was utilized to identify wells that contained a single cell. Colony formation was scored 15–20 days after initial seeding. The percentage of cells that formed spheres was determined by the following formula: 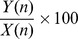 where X(n) is the number of wells in which a single cell was present and Y(n) is the number of wells in which one NS developed from a single cell. Wells containing either none or more than one cell were excluded for the analysis.

### Differentiation assay of spheres derived from one mother cell

Glioma spheres derived from one mother cell in the subsphere-forming assay were mechanically disaggregated into single cells that were seeded into standard medium permissive for differentiation (without growth factors and supplemented with 10% of FBS) for 1–45 days. Then, cells were analysed at different time points.

### Antibodies

To evaluate the expression of stemness and differentiation markers, the following antibodies were used: anti-CD133 (clone AC133 PE conjugated) (Miltenyi Biotec, Bergisch Gladbach, Germany); anti-Nestin (Chemicon, Tamecula, CA, USA); anti-Sox2 monoclonal, (R&D System, MAB2018); anti-Musashi-1 (monoclonal, R&D System, MAB2628); anti-Galactocerebroside (GalC, monoclonal IgG3, Millipore Corp., Bedford, MA, USA); anti-Glial Fibrillary Acid Protein (GFAP, monoclonal, IgG3, Millipore), anti-Neuronal Class IIIβ-Tubulin (monoclonal IgG2a, TUJ1 Clone, Covance Research Group Inc.).

### Flow cytometry

To evaluate CD133 expression by flow cytometry, cells were mechanically dissociated, washed twice in a solution of ethylenediaminetetraacetic acid (EDTA) 2 mM in phosphate-buffered saline (PBS) containing 0.5% bovine serum albumin (BSA), and then incubated with anti-CD133/1 (AC133)-PE antibody (Miltenyi Biotec, Germany) 1∶20 for 30 min. at 4°C in the dark. Cells were washed, and suspended in PBS for analysis. They were then analysed by flow cytometry in a PCA-96 System machine (Guava Technologies, Hayward, CA, USA).

The Caco-2 cell line, a continuous line of a heterogeneous human epithelial colorectal adenocarcinoma, was used as a positive control.

### Immunocytochemistry

#### Cryosectioning of NS

Whole NS were collected from culture flasks, washed with PBS to remove the excess of culture medium, and fixed in 4% ***paraformaldehyde*** (PFA) for 10 min. at room temperature. NS were then placed in a PBS/20% sucrose overnight at 4°C. They were then mounted in OCT (optical cutting temperature) compound (Sakura, Tissue Tek; Torrance, CA), followed by freezing at −80°C. Slices of 10 µm were obtained on a cryostat, and placed on slides (“Super Frost Plus”, Menzel-Gläser, Braunschweig, Germany).

Cells growing in monolayer were seeded on “Super Frost Plus” slides and, at the different culture times, they were fixed in 4% PFA for 10 min. Following washing with PBS, slides were kept at −80°C until they were processed.

For immunocytochemical analysis, after rehydration with PBS, the same procedure was followed for cryosections and coverslips. Sections were covered with blocking solution (Super block, UCS Diagnostics, Roma, Italia) for 8 min. or with 0.3% (v/v) TritonX-100, 1% (w/v) BSA and 10% (v/v) “normal donkey serum” in PBS for 1 hour. Subsequently, they were incubated overnight at 4°C with the following primary antibodies (diluted in blocking solution): anti-Nestin (1∶200); anti-Sox2 (1∶50); anti-Msi-1 (1∶200); anti-GalC (1∶100); anti-GFAP (1∶500); anti-βIII-Tubulin (1∶200). After rinsing with saline buffer, slices were incubated with the HRP polymer conjugate (SuperPicture Polymer Detection Kit, Zymed, San Francisco, CA, USA), and after a washing step, 3,3′-diaminobenzidine (Vector, Burlingame, CA, USA) was used as the chromogen. Nuclei were counterstained with Harri’s haematoxylin. Negative controls were performed by omitting the primary antibody.

### Western-blot analysis

Cells from both monolayer cultures and NS, after 0–45 days of culture, were collected in a lysis buffer (150 mM NaCl, 1% Nonidet P-40, 0.5% Triton X-100, 0.5 mM EDTA, 0.1% SDS, 50 mM Tris-HCl, pH 7.6), containing protease inhibitor cocktail (Sigma-Aldrich), and 17.4 µg/ml phenylmethylsulfonyl fluoride (Sigma-Aldrich). Protein extracts were quantified by Bradford Protein Assay. For Western-blot analysis, 50 µg of total proteins were resolved on denaturating polyacrylamide gels (SDS-PAGE): 10% (Msi-1, βIII-Tubulin) and 8% (Nestin) and transferred to polyvinylidene difluoride (PVDF; Immobilon P, Millipore, USA) membrane by electroblotting. Membranes were blocked with PBS-T (PBS buffer saline with 0.1% Tween-20) containing 5% BSA and then incubated overnight with primary antibodies. After washing for 3×5 min., membranes were probed 1 h at room temperature with secondary antibody (anti-mouse IgG peroxidase conjugate, Sigma, 1∶10,000). The revelation was performed by chemiluminescence.

The signals were quantitated by densitometric scanning (ChemiDoc documentation system/QuantityOne quantitation software; Bio-Rad Laboratories, Hercules, CA, USA).

### Confocal Ca^2+^ imaging

Confocal Ca^2+^ imaging was performed as previously described [32]. Briefly, cells plated on glass coverslips were incubated for 30 min. at 37°C with the Ca^2+^-sensitive fluorescent dye Fluo-4 AM (2.5 µM, Invitrogen, San Giuliano Milanese, Italy) in Tyrode’s solution containing: 150 mM NaCl, 4 mM KCl, 2 mM MgCl_2_, 10 mM glucose, 10 mM HEPES, and 2 mM CaCl_2_. The pH was adjusted to 7.4 with NaOH. The dye-loaded cells were washed and maintained on coverslips for 30 min. at room temperature (23–25°C) in fresh Tyrode’s solution to allow complete dye de-esterification. The coverslips were then transferred to a perfusion chamber placed on the stage of an inverted microscope (DM IRE2; Leica Microsystems, Wetzlar, Germany) attached to a confocal laser scanning system (TCS-SP2; Leica). Fluo-4-loaded cells were excited with the 488-nm line of an Ar/ArKr laser, and the emission signal was collected by a photomultiplier within a window ranging from 500 to 535 nm. Intracellular Ca^2+^ transients were stimulated by either cell exposure to ATP (50 µM) or membrane depolarization (obtained by exposing cells to a modified Tyrode’s solution containing 100 mM KCl). Intracellular Ca^2+^ transients induced by these stimuli were acquired at 0.5 Hz and their amplitude was measured in terms of ΔF/F ratio, i.e., 
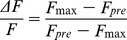
 where F_max_ represents the fluorescence recorded at the peak of Ca^2+^ transients calculated over all the recorded measure (lasting 60 s) in a region of interest traced around each cell body; F_pre_ is the average fluorescence intensity measured during the pre-stimulus period (20 s); and F_bgnd_ is the background fluorescence measured in an area of the field lacking dye-filled structures. In a subset of experiments aimed at evaluating the contribution of extra-vs. intra-cellular Ca^2+^ to the measured intracellular Ca^2+^ transients, two consecutive ATP stimulations were performed on cells at differentiation day 5. The first application of ATP was performed in normal Tyrode’s solution. After 5-min., ATP application was repeated in a modified Tyrode’s solution in which Ca^2+^ was removed (i.e. virtually Ca^2+^-free solution). Before testing the effects of Ca^2+^ removal on ATP-induced Ca^2+^ transients, the stability of Ca^2+^ responses to two consecutive ATP stimulations repeated at 5-min. interval was evaluated: differences smaller than 5% were found.

### Proliferation assay

LI cells were seeded into multiwell plates in standard medium while NS clones were seeded in NSCM (25,000 cells per well). Cell counts were determined on days 2, 4, 6, 8 and 10 after seeding using an automatic cell counter (NucleoCounter, ChemoMetec A/S, Allerød, Denmark). The medium was changed every 2 days.

The growth curve was generated so that horizontal coordinate datum defined days and vertical ordinate datum defined cell densities. Cell doubling times during logarithmic growth were calculated according to Hayflick’s formula: 
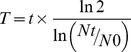
 (T = population doubling time; t = appointed time after subculture; N_t_ = number of cells at the appointed time; N_0_ = number of cells at the beginning of subculture).

A set of cell growth experiments was performed to test the effect of ATP on cell proliferation. Cells (LI, D2, F11, and D2 and F11 collected after 1-day and 5-days under differentiation medium) were seeded at a density of 50,000 cells/well in 1 ml of culture medium for 24-well plates and treated with ATP concentrations from 0 to 100 µg/ml for 0, 1, 2, 3, 4, 5 and 7 days. Cell counts were performed with a cytometer. Experiments were run in triplicate.

### *In vivo* analysis of tumorigenicity

#### Ethics Statement

All of the animals used were experimented in strict accordance with the Institutional Animal Care and Use Committee guidelines in force at the Catholic University of the Sacred Heart and every effort was made to minimize suffering. The work was carried out under the authority of the Animal Ethics Committee, “A. Gemelli”, School of Medicine, Catholic University of the Sacred Heart, Rome, Italy (approved project licence: prot.pdc.CESA/A/42/2011).

Athymic nude mice (nu/nu; 6–8 weeks old; Charles River Laboratories, Wilmington, MA, USA) were anesthetized with i.p. ketamine and xylazine, and stereotactically implanted with either isolated D2, F11 cells (10,000 or 1,000 per mouse) or LI cells (500,000 or 10,000 per mouse) in 3 µl of PBS in the right striatum. A control group received vehicle only (PBS). The implanted mice, 4 animals per group, were weighed once per week, monitored twice per week and were killed when they developed neurological symptoms (ataxia/incoordination/staggering/unbalanced). Mice were sacrificed by cervical dislocation under a deep anaesthesia. Brain sections were examined for the presence of tumors, as described below.

### Hematoxylin–eosin and immunohistochemistry staining of brain sections

The tumor-cell-implanted mice brains were fixed with 4% paraformaldehyde and cut with a microtome into coral sections. For hematoxylin–eosin staining, brain sections were mounted on slides stained with Harri’s hematoxylin for 2 min. first, and then counterstained with alcoholic eosin. To characterize the brain tissue by immunohistochemistry, sections were stained with primary antibodies for human-specific anti-Nestin (1∶200), anti-βIII-Tubulin (1∶200), and anti-GFAP (1∶500), following the same procedure described above.

### Statistical analysis

All values in the figures and text were shown as means ± SD. Any significant differences among mean values were evaluated by the Student’s t test or one-way ANOVA. A two-sided p<0.05 was accepted as significant.

## Results

### Isolation of CSC by neurosphere (NS) formation assay

**Figure 1:**
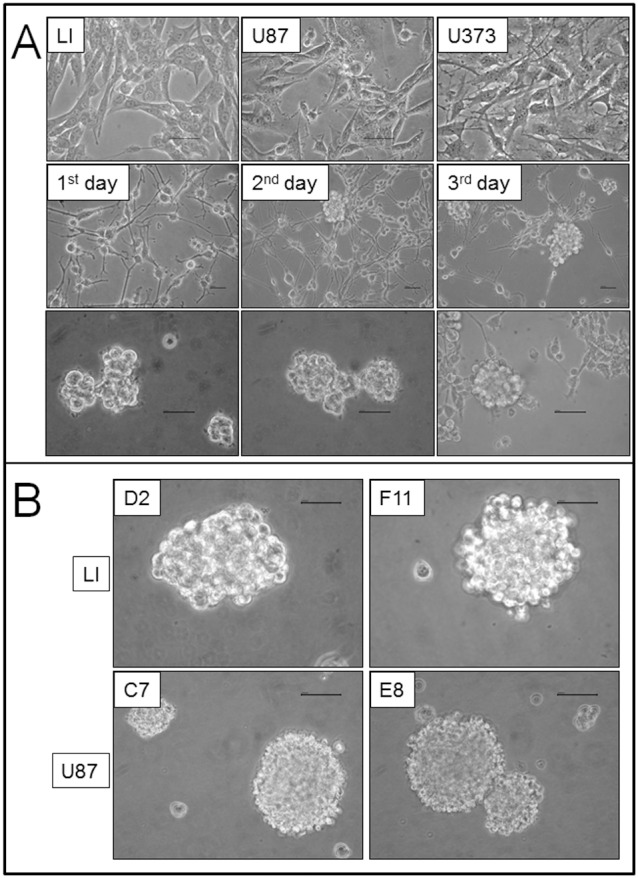
Representative phase contrast microscopy analysis of cultured cells. (A): top, parental cell lines (LI, U87 and U373 cells) cultured in their standard medium added of 10% serum. Middle, formation of neurospheres on top of the monolayers of U373 cell line. Images were taken after 1, 2 and 3 days in neurosphere medium. Bottom, images of primary NS generated by LI, U87 and U373 cells by day 8. (B) Clones derived from LI (D2 and F11) and U87 (C7 and E8) primary NS. Original magnification 200× or 400×. Scale bar = 100 µm.

Under the culture conditions for NSCM, spheres resembling typical NS rapidly formed on top of the monolayers of all cell lines (Figure 1A). About 3 days later, U87 and LI cell lines grew entirely spherically (10–20 cells) with no attached cells (primary NS), while some adherent cells persisted in U87 cell line. After 1–2 weeks of culture, the formation of tumor spheres was detected and photographed under an inverted microscope. The spheres could be propagated and continuously cultured in a free-floating form (Figure 1A). Therefore, all cell lines formed self-renewable spheres in culture.

### Limiting Dilution/Spheres Tumor Initiation (TS-CI) Analysis

For determining the ability of the three cell lines to form NS, a TS-CI analysis was performed. The frequency of TS-ICs was 1.56% for the U373 cells (1 cell every 64 showed the ability to form primary NS), 1.45%, for the U87-MG cells (1 cell every 69 showed the ability to form primary NS), and 2.74% for LI cells (1 cell every 36 exhibited the ability to form primary NS).

### Single colony formation analysis

To assess the self-renewal capacity of primary NS, a clonogenic assay was performed. The efficiency of colony formation was 9.5%, 10.5% and 33.3% for NS derived from U373, U87 and LI, respectively.

Growing clones were selected, disaggregated, and maintained over many subcultures. Two clones (D2 and F11) were obtained from primary NS derived from LI and two (C7 and E8) from U87 which formed self-renewable spheres in culture (Figure 1B). None of the clones derived from U373 was able to persist in culture even when a different medium was employed. The morphology of the clones was variable. F11, C7 and E8 grew entirely spherically, while D2 tended to form both spherical or elongated aggregates (Figure 1B).

### Expression of stem cell markers and lineage-specific markers

**Figure 2:**
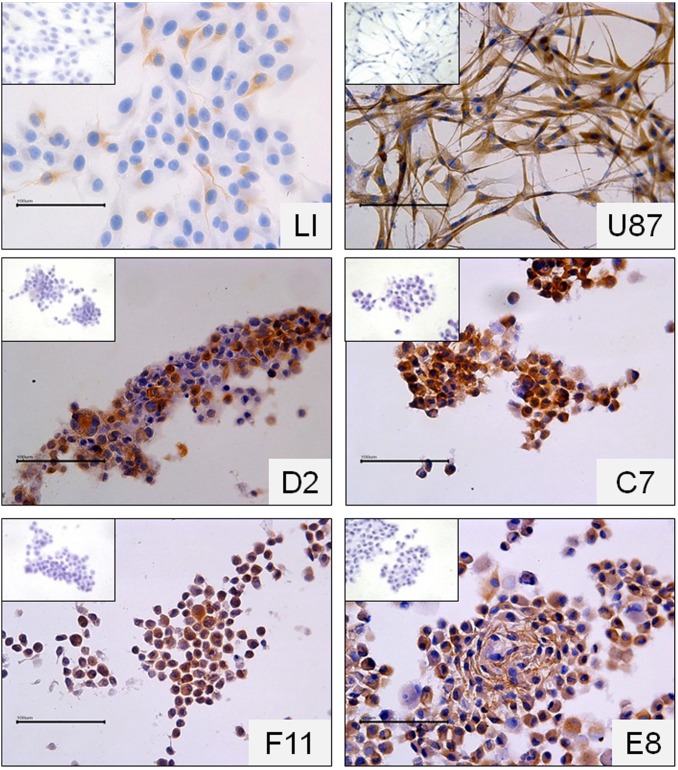
Parental cell lines and NS derived from a single mother cell were stained by stemness marker Nestin. The inserts refer to negative controls. Original magnification 400×. Scale bar = 100 µm.

CD133, Nestin, Sox2 and Msi-1 were evaluated to assess the staminal nature of selected clones. Using immunocytochemistry, 50% of LI cells and 100% of U87 were Nestin-positive and the staining was located at the cytoplasmic level. In both D2 and F11 clones derived from primary NS of LI cells, an increase in stem cell marker expression was observed. More than 80% of cells from both clones were positive for Nestin. No increase in Nestin staining was observed in both clones derived from U87 cells with respect to the parental cell line (Figure 2).

On the basis of this evidence and morphological aspect, subsequent experiments have been performed only on clones D2 and F11 with the aim of verifying if the observed differences could correspond to differences in behaviour.

**Figure 3:**
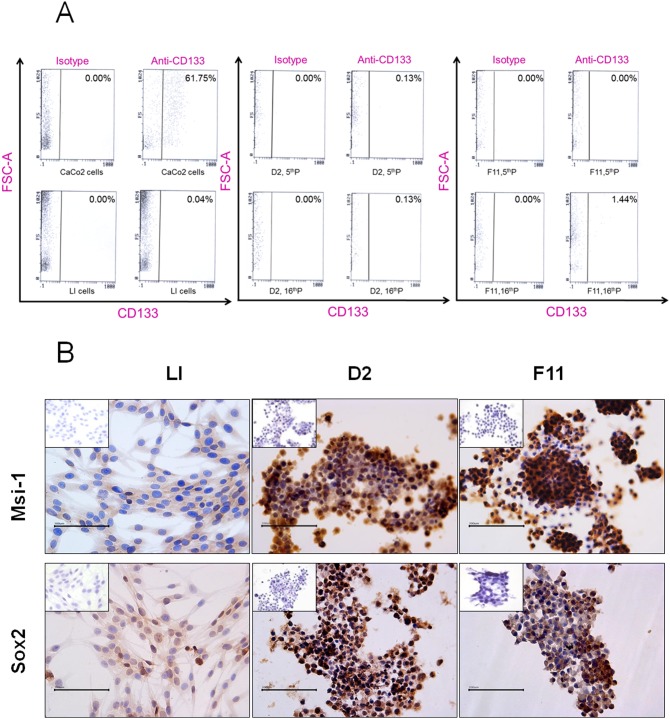
A: Flow cytometry analysis to measure CD133 expression of cells derived from LI monolayer cultures and D2 and F11 clones. CaCo_2_ cells are positive control. CD133 expression was evaluated in both clones at 5^th^ passage (5^th^P) and 16^th^ passage (16^th^P). B: Data from a representative experiment of immunocytochemical staining for Msi-1 and Sox2 expression in LI, D2 and F11 cells. The inserts refer to negative controls. Original magnification 400×. Scale bar = 100 µm.

**Figure 4:**
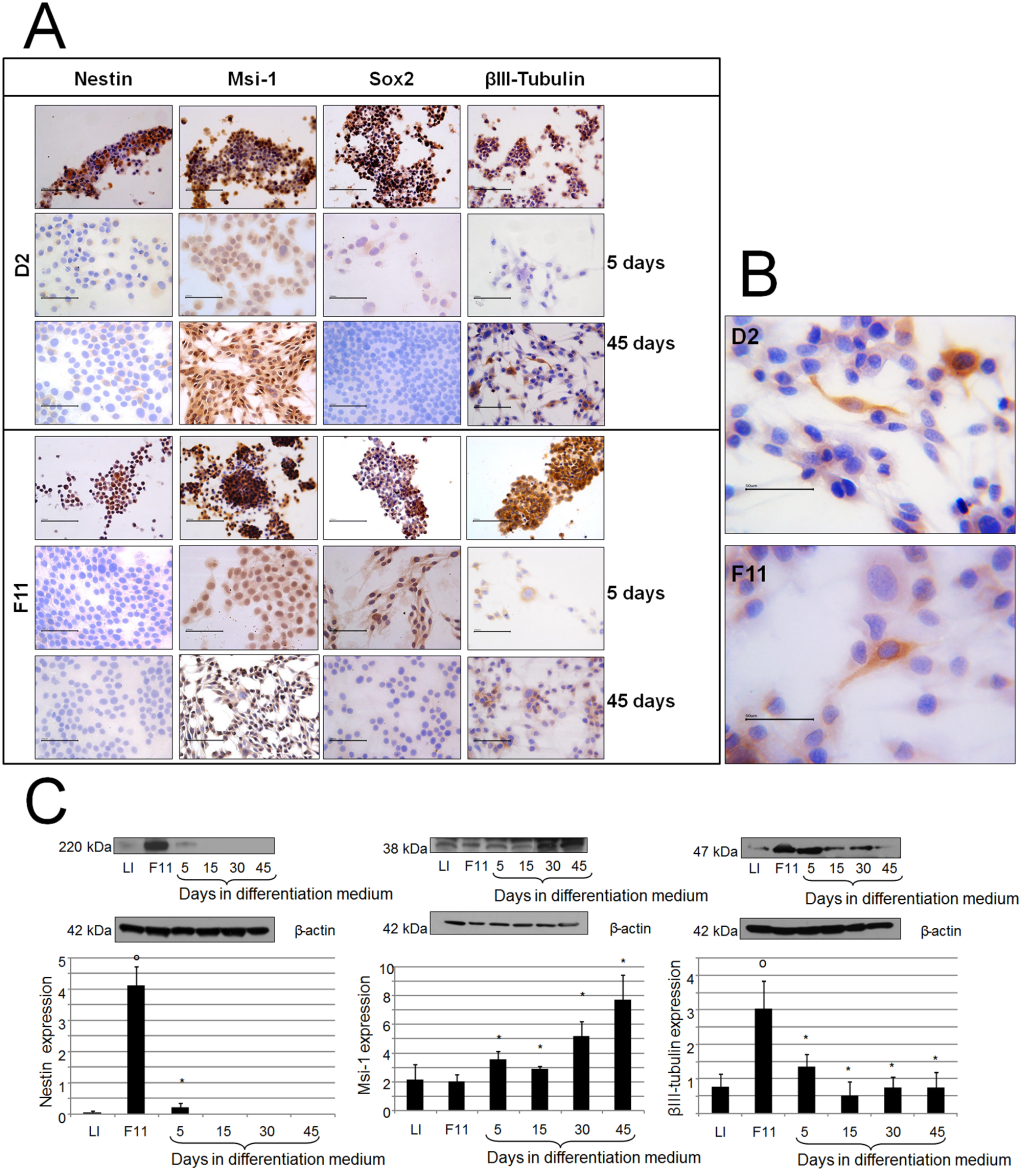
A: Immunocytochemical staining for Nestin, Msi-1, Sox2 and βIII-Tubulin expression in D2 and F11 cells cultured in neurosphere medium (without serum) or in differentiation medium (presence of serum) for 5–45 days. Original magnification 400×. Scale bar = 100 µm. B: Some βIII-Tubulin positive cells exhibiting a typical neuronal-like morphology. Original magnification 630×. Scale bar = 50 µm. C: Western blot analysis of Nestin, Msi-1 and βIII-Tubulin expression in F11 cells cultured for different times in differentiation medium. Proteins levels were analyzed by immunoblot analysis and were quantified by densitometry and standardized against the levels β-actin as a loading control. Values are the mean ± SD of tree independent experiments. Similar results were obtained from D2 cells. Immunoblottings from representative experiments are shown. *p<0.05 Student’s *t*-test vs. parental cell lines, *p<0.01 vs. F11 cells.

Analyzing CD133 expression by flow cytometry, we detected 0.04, 0.13, and 1.44% CD133 positive cells in LI, D2, and F11 cells, respectively (Figure 3A). These values persisted after various cycles of cryopreservation. Using immunocytochemistry, we found that 30% LI cells present in small groups in the monolayer culture were positive for Sox-2 that was typically localized in the nuclei; finally 100% LI cells showed weak Msi-1 cytoplasmic staining (Figure 3B). In both D2 and F11 clones, an increase of stem cell marker expression was observed. Eighty percent of cells from both clones were positive for Nestin and 100% for Sox-2. A stronger expression of Msi-1 was detected in 100% of cells (Figure 3B). Nestin expression in both clones was confirmed by Western blot analysis. Figure 4C refers to data obtained in F11 clone.

Spheres were cultured under differentiation conditions for different times and were analyzed for the expression of stem cell markers and lineage-specific markers. After overnight incubation in differentiation medium, the neurosphere cells were adherent to the culture plate and grew in monolayer. The expression of neural-specific βIII-Tubulin, GFAP, and GalC was evaluated by immunocytochemistry. Both βIII-Tubulin and GFAP were highly expressed in parental cell line and in both clones, while GalC was not expressed (data not shown). Upon differentiation conditions, the expression of neural-specific βIII-Tubulin decreased (Figure 4A), while that of GFAP did not change significantly (data not shown). Notably, some of βIII-Tubulin expressing cells had a neuron-like morphology (Figure 4B), while others were multinucleated cells.

The decrease in βIII-Tubulin expression was also assessed by western blot analysis in both D2 (data not shown) and F11 (Figure 4C) clones.

In D2 and F11 clones a decrease in both Nestin and Sox-2 immunopositivity until undetectable levels was observed after longer lasting NS cell culture in differentiation medium (Figure 4A). The striking reduction of Nestin levels was further confirmed by Western blot analysis (Figure 4C refers to data obtained in F11 clone). No apparently variation in the Msi-1 expression, analysed by immunocytochemistry, was observed, but its location changed between nucleus and cytoplasm depending on the time of permanence in serum-containing medium (Figure 4A). Western blot analysis demonstrated that LI cells and both clones exhibited a similar expression of Msi-1, while cells from both clones under differentiation showed a higher level of the protein. Figure 4 shows data obtained in F11 clone.

### Confocal Ca^2+^ imaging

**Figure 5:**
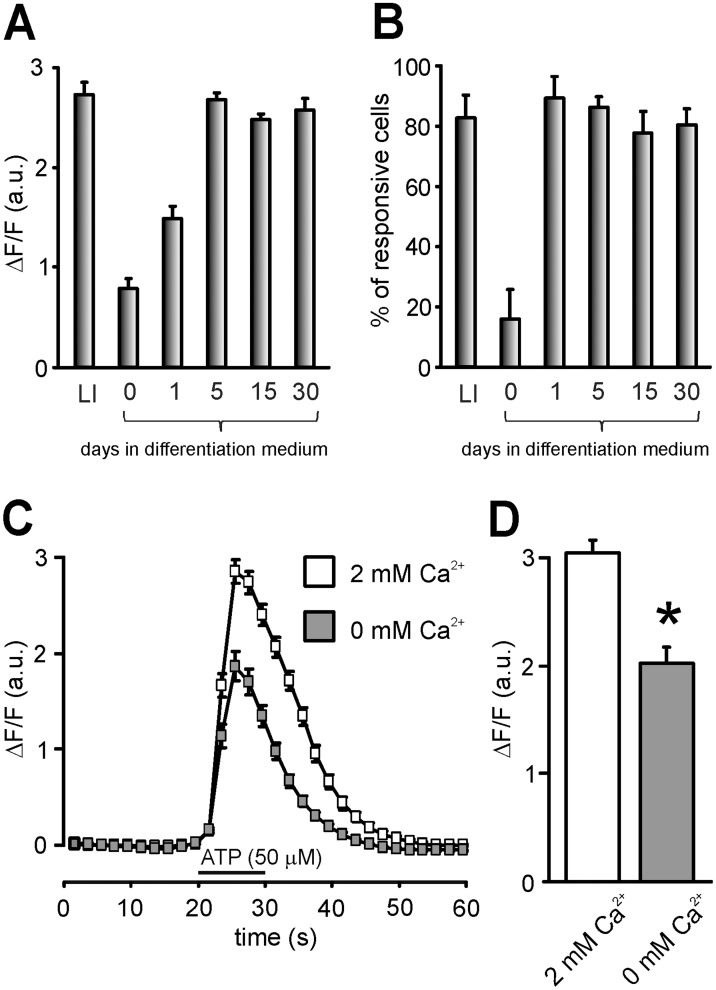
(A) Bar graph representing the mean amplitude at the peak of ATP-induced Ca^2+^ signals (expressed in terms of the ΔF/F ratio) at different points of permanence of CSC in differentiation medium. Extracellular application of 50 µM ATP induced intracellular Ca^2+^ transients in CSC whose fluorescence increased with the time of differentiation reaching the maximum in cells cultured for 5 days. Since 5^th^ day fluorescence remained constant. (B) Bar graph representing the percentage of cells responsive to ATP (50 µM) with an increase in intracellular Ca^2+^. (C) Comparisons of the intracellular Ca^2+^ transients induced by 50 µM ATP in cells at 5^th^ day differentiation in the presence (white) and in the absence (gray) of extracellular Ca^2+^. (D) Bar graph quantifying the mean amplitude at the peak of intracellular Ca^2+^ transients in the presence (white) and in the absence (gray) of extracellular Ca^2+^. *p<0.001.

In undifferentiated cells (referred as time-point day-0), KCl stimulation produced Ca^2+^ transients in less than 10% of total cells (*n* = 9 out of 127), and the mean amplitude of these transients was 0.36±0.02. A low responsiveness to depolarizing stimuli was also observed in differentiating cells. In fact, the percentage of cells responding to KCl stimulation with Ca^2+^ transients was lower than 10% at day-5, day-15 and day-30 too, with mean amplitudes of 0.75±0.25 [*n* = 7 out of 287], 0.42±0.07 [*n* = 10 out of 418] and 0.34±0.02 [*n* = 7 out of 418], respectively. These results indicate that LI-derived human stem cells, either undifferentiated or at different stages of differentiation, do not exhibit functional voltage-gated Ca^2+^ channels. However, when these cell preparations were exposed to 50 µM ATP, clearly detectable intracellular Ca^2+^ transients were observed. In undifferentiated cells (day 0), ATP produced Ca^2+^ signals in 16.1±9.8% of total cells (39 out of 127) with mean amplitude of 0.79±0.10. After 1 day culture in the differentiation medium, the percentage of responding cells rose to 89.2±7.2% (*n* = 115 out of 132) and the mean amplitude of transients was similarly increased (1.49±1.13). At day-5, no further increase in the percentage of responding cells was observed (86.3±3.5%; *n* = 430 out of 492) but the transient’s amplitude reached a maximum value of 2.69±0.07. No significant changes in Ca^2+^ signal amplitudes were observed during the following days of differentiation up to 30 days: the ΔF/F values being 2.48±0.07 at day-15 (77.8±7.1% of responding cells; *n* = 334) and 2.55±0.11 at day-30 (80.5±5.1% of responding cells; *n* = 306). Interestingly, similar results were obtained when LI cells (the cell line from which stem cells were derived) were exposed to 50 µM ATP (ΔF/F = 2.72±0.12; 79% of responding cells; *n* = 256; one-way ANOVA: F_(3,1165)_ = 1.49; *P*>0.05) (Figure 5A–B).

To determine whether ATP-induced intracellular Ca^2+^ transients in differentiating stem cells depended on either Ca^2+^ entry from extracellular medium through the ionotropic purinergic receptors P2X or Ca^2+^ release from intracellular stores following activation of the metabotropic receptors P2Y, a subset of experiments was carried out in cells at day-5 in which stimulation with 50 µM ATP was performed in the presence and absence of extracellular Ca^2+^. The removal of Ca^2+^ from extracellular medium reduced the amplitude of ATP-induced Ca^2+^ transients by 38.4±3.9% (from 2.96±0.11 to 1.93±0.15; *n* = 127; paired *t*-test: *P*<0.0001) (Figure 5C–D).

### Proliferation assay

The doubling time (DT) of LI cells cultured in their standard medium, determined from the growth curve, was about 36 hours. The DT of D2 and F11 was 70.5 and 88 hours at a low passage (5^th^) and 66.1 and 69.2 at a high passage (16^th^) when cultured in NSCM. The DT of CSC decreased at increasing passages in culture. The DT of both clones in medium with 10% FBS decreased compared with that of CSC cultured in NSCM and increased at high passages in culture. However, the DT of CSC in standard medium was longer than that of the parental cell line LI (36 h) (Table 1).

**Table 1:** Doubling time of D2 and F11 clones cultured in NSCM (serum-free; presence of growth factors) or in medium with 10% FBS added.

	D2		F11	
Passage number	serum free	10% FBS	serum free	10% FBS
**5^th^**	70.5*	42.5	88.0	43.3
**16^th^**	66.1	56.0	69.2	51.1

*hours.

None of the concentrations of ATP tested had a significant effect on cell proliferation (data not shown).

### *In vivo* growth of GBM spheres

To check whether clones or LI cells were tumorigenic in vivo, cells were xenografted into the striatum of nude mice. LI cells were able to generate tumors after implantation of 500,000 (4 of 4 mice) or 10,000 (1 of 4 mice) after about 10 and 30 weeks, respectively. One thousand or 10,000 D2 cells, and 1,000 or 10,000 F11 cells formed tumors (3 of 4 mice) after about 21 and 24 weeks, respectively (Table 2). The morphology of tumors varied. In some cases tumors grew well circumscribed, in others they were highly infiltrative and grew diffusely. Occasionally necrotic foci were detected, but no true pseudopalisading was found.

**Table 2:** Tumorigenicity of LI cells and CSC clones (D2 and F11).

Cell type	Number of injected cells	Weeks after injection									Tumor incidence
		10	12	14	16	18	20	22	24	>46	
**D2**	10,000							1	2		3/4
**D2**	1,000						1	1	1		3/4
**F11**	10,000							2	1		3/4
**F11**	1,000							1	2		3/4
**LI**	500,000	1*	2			1					4/4
**LI**	10,000						1				1/4
**Vehicle** **	0										0/4

*Number of animals sacrificed.

**Phosphate buffered saline.

**Figure 6:**
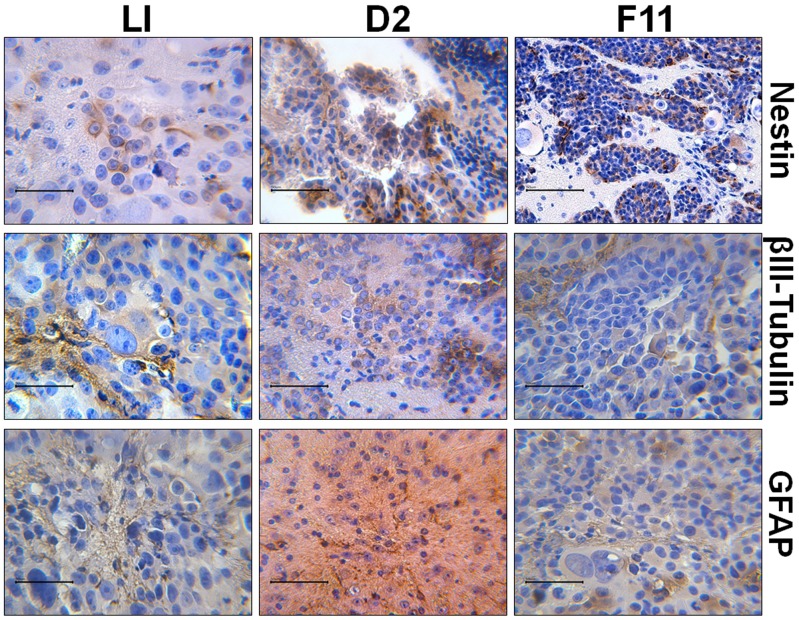
Immunostaining of brain sections from mice implanted with 500,000 LI cells, 10,000 D2 cells and 10,000 F11 cells. Original magnification 630×. Scale bar = 50 µm.

Tumor sections were immunostained for Nestin, βIII-Tubulin and GFAP. All antigens were detected in all tumors analysed and were diffusely present throughout the tumors (Figure 6).

## Discussion

In this study, we provided evidence that a subpopulation with morphological and behavioural features of CSC can be obtained from three established glioma cell lines with different degrees of malignancy. The clones derived from LI cell line, D2 and F11, showed different morphology. This is in agreement with findings previously reported by some Authors, who suggested that different phenotypes of CSC exist in glioma [5], [12].

Nowadays, CD133 remains the most studied marker [45], although recent works questioned its role [46]. Nevertheless, some Authors have reported the prognostic role of CD133 [47]–[49]. In particular, Liu et al. suggested that CD133^+^ cells in GBM are indeed responsible for the tumor propagation and vascularization, and that they are capable of shifting to a mesenchymal phenotype [49].

In both clones, D2 and F11, an increase in the fraction of CD133^+^ cells compared to the LI parental cell line was observed. The percentage, although small (<2%), of CD133^+^ cells in D2 and F11 clones is in the range reported by some Authors [16], [20], but different from that found by others [37]. After many months, in clones that had undergone many passages in culture and had been subjected to various cycles of cryopreservation, a CD133 expression superimposable to that of the first evaluation was detected. This indicates no variation in the population of both clones.

The expression of markers other than CD133 (Nestin, Sox2, Msi-1) was evaluated by immunocytochemistry on cryosections of embedded D2 and F11 clones, following the method described by Bleau et al. [50].

Both clones were found strongly positive for all the markers evaluated, in agreement with data in the literature [7], [12], [18]. The expression of these markers was higher than in the parental cell line. Increased expression of Nestin was confirmed by Western blot analysis.

These results are indicative of stem cell-like properties of isolated clones. In fact, Nestin and CD133 are generally regarded as markers of both neural stem cells and CSC, and characterize the pathological glioma stem cell niches [51]. Sox2 has been more consistently associated with multipotentiality and progenitor cell proliferation, and it is essential for glioma-initiating cells to retain their stemness [52].

The ability to differentiate is another typical feature of CSC and it is generally induced by maintaining the cells in medium supplemented with 10% serum [12], [16]–[23]. In this condition, Nestin and Sox2 decreased dramatically in both D2 and F11 cells. These findings are in agreement with other reports showing that Nestin and Sox2 are down-regulated in the process of terminal neuronal differentiation [53], [54].

As far as Msi-1 is concerned, this is a complex multifunctional protein crucial for the maintenance of the stemness state in normal tissues, and strongly expressed together with Nestin in highly invasive tumors [55], [56] as well as in reactive astrocytes and in cells that differentiate into astrocytes [57]. Msi-1 cell localization changed during clone maintenance in differentiating medium from cytoplasm to nucleus. This is in agreement with other Authors who have demonstrated a nuclear localization of the protein in murine PC12 pheochromocytoma cells during neuronal differentiation [58].

It has been reported that CSC share with normal stem cells the capability to differentiate into specialized cell types [1]. The markers generally used to evaluate the differentiation in neural stem cells are βIII-Tubulin, GFAP and GalC [9], [12], [16]–[18], [21]–[23].

Neither D2 nor F11 cells after permanence in serum containing medium expressed GalC. This is consistent with data in the literature which indicate that the ability of CSC to differentiate into oligodendrocytes is almost null [9] or very limited [12], [23], even when markers other than GalC have been considered [16]. On the contrary, both D2 and F11 cells expressed GFAP and βIII-Tubulin at high levels. These results are in agreement with recently published data [7], [24].

The expression of GFAP did not significantly change in our cells after culturing in serum-containing medium, while that of βIII-Tubulin decreased. Although neuronal differentiation results in increased βIII-Tubulin levels, a high expression of the protein has been described in high-grade astrocytomas [59]. Therefore, the decrease in βIII-Tubulin we observed might be suggestive of a more differentiated phenotype of CSC, whose maturation process is, however, reported to be incomplete [17]. Interestingly, in both clones, some elements positive for βIII-Tubulin show a neuronal morphology.

The absence of functional voltage-dependent Ca^2+^-channels in differentiating cells confirm that our clones are unable to differentiate in neurons. In fact, it has been reported that NSC differentiation is strongly correlated with the expression of voltage-gated Ca^2+^ channels [31], [32]. However, Ca^2+^ is required by GBM cells as a second messenger to support cell migration and oscillatory changes in intracellular Ca^2+^ concentration correlate with cell invasion and migration [33]. Interestingly, ATP dependent Ca^2+^ signals have been detected in cells cultured under differentiation conditions, while they are not expressed by NS cells. This is important, considering that one property of astrocytes is their responsiveness to ATP. ATP-mediated signaling has been shown to be relevant in the differentiation of the murine embryonal carcinoma cell line P19 [57], [61]. Moreover, it reduces tumor sphere growth and the number of CD133^+^ stem cell population in GBM cells [35]. Our data suggest that early stages of CSC differentiation correlate with the generation of intracellular Ca^2+^ signals mediated by the activation of ATP receptors, especially the metabotropic P2YRs. Nevertheless, no significant variation in cell growth was induced by ATP treatment in our models.

The CSC nature of both clones, D2 and F11, is proved by their tumorigenicity. When cells were orthotopically inoculated in the striatum of immunosuppressed mice, the occurrence of tumors with histopathologic features comparable to GBM was observed. The histological heterogeneity of tumor is consistent with reports in the literature [18], [40]. It is interesting to note that tumors have arisen in the time range reported by other Authors [7], [12], [18] and that the concentration of NS cells required to induce them is 50-fold less than that required for LI cells. Moreover, tumors in xenografts have maintained the characteristics of the original cells about expression of Nestin, GFAP and βIII-Tubulin.

## Conclusions

All results collected so far confirm the possibility of isolating cells from established human glioma cell lines, with specific characteristics of stemness and with tumorigenic properties. Both clones derived from LI, D2 and F11, show the same characteristics and, at present, we conclude that, despite the initial evidence of the morphological diversity of the neurospheres, the cells that compose them are homogeneous for the expression of stem cell markers as well as for their behaviour regarding the differentiation, and for their tumogenicity. In our opinion, both clones might be used for further studies concerning the possibility of deepening the existing knowledge about CSC in GBM and testing new drugs which may be more effective in GBM treatment. The availability of many CSC models, on the basis of inter- and intra-tumoral cancer initiating cell heterogeneity, will be essential to fully translate the basic research findings into clinical trials, as recently suggested by Pointer et al. and Romaguera-Ros et al. [4], [5].
