# Correction: Isolation of Cancer Stem Cells from Three Human Glioblastoma Cell Lines: Characterization of Two Selected Clones

**DOI:** 10.1371/journal.pone.0114081

**Published:** 2014-11-14

**Authors:** The PLOS ONE Staff

There is an error in the funding statement. Please refer to the correct funding statement below.

This work was supported by a grant from the Catholic University of the Sacred Heart "Progetti Fondi di Ateneo". The funders had no role in study design, data collection and analysis, decision to publish, or preparation of the manuscript.
